# Strategies to Improve Health Technologies for Older Adults With Cognitive Impairment and Dementia

**DOI:** 10.1093/geroni/igaa057.3179

**Published:** 2020-12-16

**Authors:** Stacey Schepens Niemiec, Elissa Lee, Jeanine Blanchard, Adam Strizich

**Affiliations:** University of Southern California, Los Angeles, California, United States

## Abstract

Technology may improve health self-management of older people with cognitive deficits, yet these individuals may have unique needs influencing its utility. This study’s purpose was to gather current strategies described in the literature and by expert stakeholders for utilizing digital health technologies in older adults with cognitive impairment (CI) or dementia. We conducted a rapid literature review to identify articles that featured digital health technology use in persons with CI/dementia. Additionally, we conducted interviews (n=12) with expert stakeholders who were identified through online academic, professional, and community organization biographies and snowball referral. Qualitative-based thematic analysis was used to identify emergent themes from selected literature and transcribed interviews. Recommended strategies addressed instructional methods (e.g., reducing distractions), technology adaptations (e.g., simplified interface), care partner involvement, and dosage/exposure. Findings are applicable to development of technology-driven interventions and products, with the aim of improving the effectiveness of such technology for older people with CI/dementia.

